# Identification and characterization of LuxR solo homolog PplR in pathogenic *Pseudomonas plecoglossicida* NB2011

**DOI:** 10.3389/fcimb.2024.1458976

**Published:** 2024-10-25

**Authors:** Shanshan Li, Tingting Jia, Yu Chi, Jigang Chen, Zhijuan Mao

**Affiliations:** Biological and Environmental College, Zhejiang Wanli University, Ningbo, China

**Keywords:** *Pseudomonas plecoglossicida*, LuxR solo, AHLs, mutant, biological phenotypes

## Abstract

*Pseudomonas plecoglossicida* is a causative agent of visceral granulomas in large yellow croaker (*Larimichthys crocea*). Quorum sensing (QS) is widely involved in imparting virulence to pathogenic bacteria; however, it has not been studied in *P. plecoglossicida*. In this study, we annotated a LuxR family transcriptional regulator in *P. plecoglossicida* NB2011 and designated as PplR. We aligned the protein sequence by BlastP and Clustal X2, monitored the N-acyl-homoserine lactone (AHL) signal production through cross-feeding bioassay and HC-MS/MS; investigated exogenous AHL signal binding by recombinant expression and thin layer chromatography; constructed a deletion mutant of the target gene by method of double homologous recombination; sequenced the transcript RNA and analyzed the data; additionally, characterized phenotypes of wild type and mutant strain. The LuxR homolog PplR was found to share high similarity with PpoR—the LuxR solo of *Pseudomonas putida*—without a cognate LuxI. The wild-type strain did not produce any AHL signals and the recombinant LuxR protein was found to bind C6-L-homoserine lactone (C6-HSL), C8-HSL, 3-oxo-C10-HSL, and 3-oxo-C12-HSL. RNA-seq analysis indicated 84 differentially expressed genes—5 upregulated and 79 downregulated—mainly enriched in gene ontology terms, such as flagella-dependent motility, integral component of membrane, DNA binding and transcription, and metal ion binding, suggesting that PplR is a master transcription regulator. The mutant strain showed attenuated biofilm-forming ability and stress resistance, and the data support a role for PplR in the regulation of these traits in *P. plecoglossicida* NB2011 independent of the presence of AHL signals. This is the first study to provide QS-related information on *P. plecoglossicida*.

## 1 Introduction

Quorum sensing (QS)—chemical-based signaling—is a prevalent cell-to-cell communication system in bacteria, which regulates gene expression in response to cell density. In Gram-negative bacteria, this system comprises signal molecular synthase, signal receptors, and various response regulators (Waters and Bassler, 2005). N-acyl-homoserine lactones (AHLs) are the most common QS signal molecules in Gram-negative bacteria. The first identified QS system is the “LuxI/LuxR” system in *Vibrio fischeri*. In this system, an autoinducer, namely, 3-O-C6-homoserine lactone, is produced by LuxI, which is recognized by a cytoplasmic autoinducer receptor LuxR, a DNA-binding transcriptional activator, and a control circuit of the luciferase operon (*luxCDABE*) (Stevens and Greenberg, 1997). Several LuxR receptors without a cognate LuxI, known as orphan LuxR or LuxR solo, have been found in the genomes of Gram-negative bacteria, such as QscR in *Pseudomonas aeruginosa* (Ding et al., 2018), SdiA in *Escherichia coli* and *Salmonella enterica* (Smith et al., 2011), and PpoR in *Pseudomonas putida* (Subramoni and Venturi, 2009; Fernandez-Pinar et al., 2011). QscR is the third LuxR family receptor in *P. aeruginosa*, which combines AHLs produced by the first QS system of the bacteria and suppresses the initiation of QS regulation (Chugani and Greenberg, 2014). SdiA of enteric pathogens recognizes AHLs released by environmental bacteria, being involved in the regulation of various phenotypes including biofilm formation, motility, antibiotic resistance, and virulence (Smith et al., 2011; Pacheco et al., 2021). PpoR is conserved in *P. putida* strains irrespective of the presence of AHL signals. It has been demonstrated to bind the AHL signal N-(3-oxohexanoyl)-L-homoserine lactone (3-oxo-C6-HSL) (Subramoni and Venturi, 2009); however, *P. putida* KT2440 protein has been shown to modulate competitive fitness and surface motility of the bacteria independent of the AHL molecules (Fernandez-Pinar et al., 2011).

*Pseudomonas plecoglossicida* NB2011 is a causative agent of visceral granulomas in large yellow croaker (*Larimichthys crocea*) (Mao et al., 2013a). The genome of this strain (ASJX00000000) harbors a LuxR family transcription regulator. Presently, the information available on the QS system of *P. plecoglossicida* is scarce. In this study, the predicted *luxR solo* in *P. plecoglossicida* NB2011 was deleted by double homologous recombination to construct a mutant Δ*luxR* NB2011. RNA transcription analysis was conducted to identify regulatory genes; AHL detection and binding assays were performed, and the biological phenotypes of the mutant were characterized. The study findings provide some insights into the regulatory role of this protein and a better understanding of the QS system in *P. plecoglossicida* NB2011.

## 2 Materials and methods

### 2.1 Bacterial strains and plasmids

*P. plecoglossicida* NB2011 was isolated and identified (Mao et al., 2013a). *E. coli* BL21 (DE3) was used for the expression of LuxR solo. *Agrobacterium tumefaciens* KYC55 and *Chromobacterium violaceum* CV026 strains were used for AHL signal detection. *P. aeruginosa* ATCC27853 was used for AHL production. Plasmid pSmart-I was obtained from Sangon (Sangon Biotech Co., Ltd., Shanghai, China). The suicide plasmid pK18mobsacB-Ery was kindly offered by Dr. Xiaoxue Wang (South China Sea Institute of Oceanology, Guangzhou, China).

### 2.2 Sequence annotation and alignment

The sequence was annotated and adjacent genes were predicted using the JGI database (https://img.jgi.doe.gov/). Amino acid sequence similarity of LuxR homologs was analyzed by using the BlastP algorithm. The NCBI protein database was used as the source of sequences. Homologous sequences were aligned using Clustal X2 (Larkin et al., 2007). Conserved regions were searched from the aligned amino acid sequence of the LuxR family.

### 2.3 AHL detection through bioassay

The AHL signal molecules produced by bacteria were measured through cross-feeding bioassay as previously reported (Ravn et al., 2001). The engineered *A. tumefaciens* KYC55 transformed with the plasmid pJZ384, pJZ410, and pJZ372 (Zhu et al., 2003) and *C. violaceum* CV026 (McClean et al., 1997) were used for the detection of medium-/long-chain and short-chain AHL signal molecules, respectively. To test the presence of AHL molecules in the culture broth, the test and reporter strains CV026/KYC55 were parallel lined on the Luria Bertani (LB) agar medium. Purple or blue coloration was observed on Petri plates after 24 and 48 h. When the engineered KYC55 was used, isopropyl-β-D-thiogalactopyranoside (IPTG) was added (final concentration, 0.6 mM) and 10 mg/mL of 5-bromo-4-chloro-3-indolyl β-D-galactopyranoside (X-gal) was spread on the LB agar medium.

### 2.4 AHL extraction and analysis of AHL profiles by HPLC-MS/MS

*P. plecoglossicida* NB2011 was cultured overnight in LB broth in an incubator shaker at 28°C and 80 rpm. Then, 200 mL of culture supernatant was collected by centrifugation (5,000 rpm, 6 min). AHL molecules were extracted from the supernatant as previously described (Ravn et al., 2001). In brief, the supernatant was extracted with equal volume of acidified (0.1% glacial acetic acid) ethyl acetate. The treatment was replicated thrice. The extract then was dried in a rotary evaporator placed in a water bath set at 50°C. The residue was re-dissolved in 1 mL of acidified ethyl acetate and stored at −20°C until further use. Likewise, culture supernatants of *P. aeruginosa* ATCC27853 were extracted, concentrated, and stored at −20°C until further use.

The extract was suspended in 99.9% HPLC-grade methanol and injected into the LC-MS/MS spectrometer (Acquity™, Waters Corporation, Milford, MA, USA) using a BEH C18 column (50 mm × 2.1 mm × 1.7 μm) (Waters Corporation, Milford, MA, USA). Nine synthetic AHL molecules and their oxo-derivatives with known carbon chain lengths were used as standards. The synthetic AHL molecules, namely, N-butanoyl-L-homoserine lactone (C4-HSL), N-hexanoyl-L-homoserine lactone (C6-HSL), 3-oxo-C6-HSL, N-octanoyl-L-homoserine lactone (C8-HSL), 3-oxo-C8-HSL, N-decanoyl-L-homoserine lactone (C10-HSL), 3-oxo-C10-HSL, N-dodecanoyl-L-homoserine lactone (C12-HSL), and 3-oxo-C12-HSL, were purchased from Sigma-Aldrich (Merck, KGaA, Darmstadt, Germany).

### 2.5 Expression of *P. plecoglossicida* NB2011 PplR in *E. coli* BL21

The genomic DNA of *P. plecoglossicida* NB2011 was used as a PCR template. The cloning primers were designed as follows: P1 primer, 5′CGCGGATCCATGCCGCTCTGGACTCAC3′; P2 primer, 5′CCCAAGCTTTCAGATCAGGCCACGCAT3′. The amplified fragment was ligated with the *BamH*I–*Hind*III restriction sites of pSmart-I. The ligation mix was then transformed into *E. coli* BL21, thereby making it the expression strain *E. coli*/pSmart-*luxR*. The expression strain was cultured in LB broth containing 25 μg/mL kanamycin until OD_600_ (optical density at 600 nm) reached 0.6 and IPTG (final concentration 0.6 mmol/L) was then added to induce the expression of the targeted protein for 4 h at 37°C. The total culture broth before induction, culture supernatant after induction, sonicated cell supernatant, and cell residues were collected as samples for further analysis. The samples were boiled in 10× loading buffer for 10 min before being loaded onto 12% gel for protein separation by SDS-PAGE.

### 2.6 AHL binding assay

The *E. coli* BL21 transferred with plasmids pSmartI-*pplR* or pSmartI were cultured at 37°C to an initial OD_600_ = 0.1. These cultures (5-mL aliquots) were then supplemented with AHL molecules (final concentration, 10 μM) and incubated at 37°C until OD_600_ = 0.6. The protein expression was induced by the addition of IPTG (final concentration, 0.6 mM), and the cultures were further incubated for 4 h. OD_600_ was measured and equal numbers of cells were collected and treated as follows: the cell pellets were washed with PBS thrice and then re-suspended in equal volume of PBS. Thereafter, suspensions were extracted with the same volume of ethyl acetate–0.1% acetic acid as the culture volume. The extracts were then dried, re-suspended in ethyl acetate, and analyzed by thin-layer chromatography (TLC) using C18 reverse-phase chromatography plates (Shaw et al., 1997). The AHL standards with short/medium chain, including C4-HSL, C6-HSL, 3-oxo-C6-HSL, C8-HSL, and 3-oxo-C8-HSL, were detected by overlaying the TLC plate with a thin layer of LB top agar seeded with *C. violaceum* CV026; the long-chain standards including C10-HSL, 3-oxo-C10-HSL, C12-HSL, and 3-oxo-C12-HSL were detected similarly, but with LB top agar containing 100 μg/mL X-gal seeded with *A. tumefaciens* KYC55.

### 2.7 Construction of deletion mutant Δ*pplR*

Double homologous recombination was used to construct an ORF-deletion mutant of the target gene with the recombination system of plasmid pK18mobsacB-Ery, which encodes a counter selectable marker sacB (Wang et al., 2015). The primers used are listed in **Table 1**. Briefly, *pplR* upstream (529 bp) and downstream (585 bp) flanking regions were amplified by PCR with primers pairs MF1/MR1 and MF2/MR2, respectively. The upstream and downstream fragments were then linked by fusion PCR with primers MF1/MR2. The resulting fragment was ligated to the plasmid pK18mobsacB-Ery and transferred to *E. coli* DH5α. The transformed cells were selected on the LB agar medium containing kanamycin (25 μg/mL). Then, the recombinant *E. coli* was conjugated with *P. plecoglossicida* NB2011 using the counter selecting medium containing 20% sugar. The deletion of the target gene was confirmed by PCR and DNA sequencing.

**Table 1:** Primers used for deletion mutant construction.

Name	Sequence (5′-3′)	Length (bp)
MF1	GCTCTAGAAGGGTCAGGAAGCCGAAT	529
MR1	GGAATGGCTGTCAGATCAGCATCCGTTACTCCCTTGGT	
MF2	ACCAAGGGAGTAACGGATGCTGATCTGACAGCCATTCC	585
MR2	CCCAAGCTTGTACTTCTCCAGCAGGTC	
wS	CACCGAATACACCAGCTTGAT	714/1,410
wA	GGTACGCGATTCTACAGCG	
dS	CCGCAAGCTGAACAGGTT	1,610/914
dA	CTTCTTCTGAGCCAGCGA	

MF1/MR1 and MF2/MR2 primer sets were used for amplification of upstream and downstream homologous fragments of *Pseudomonas plecoglossicida* NB2011, respectively; ws/wA and dS/dA were used as detection primer sets for to amplify outside and inside regions of the target sequence, respectively.

### 2.8 RNA-seq and transcription analyses

RNA-seq analysis of *P. plecoglossicida* NB2011 (WT) and the mutant strain Δ*pplR* was performed. The WT and mutant strains of *P. plecoglossicida* were incubated in LB broth at 28°C for 24 h. Total RNA was extracted using TRIzol^®^ reagent according to the manufacturer’s instructions (Invitrogen) and genomic DNA was removed using DNase I (TaKara). Then, the RNA quality was determined by 2100 Bioanalyzer (Agilent) and quantity was determined by ND-2000 nanodrop (NanoDrop Technologies). Only high-quality RNA samples (OD_260/280_ ≥ 1.8, OD_260/230_ ≥ 1.0, RIN ≥ 6.5, 28S:18S ≥ 1.0) were used to construct the RNA sequencing library. RNA-seq transcriptome library was prepared with the TruSeq™ RNA sample preparation kit from Illumina (San Diego, CA) using 2 μg of total RNA. High-throughput sequencing was performed using the Illumina Novaseq platform. The processing of original images to sequences, base-calling, and quality value calculations were performed using the Illumina GA pipeline (version 1.6), through which 150-bp paired-end reads were obtained. The data generated from the Illumina platform were used for bioinformatic analysis. All the analyses were performed using the free online platform of the Majorbio Cloud Platform (www.majorbio.com) from Shanghai Majorbio Bio-pharm Technology Co. Ltd. The reference genome was *P. plecoglossicida* NB2011 (GenBank: ASJX00000000). Genes were considered to be significantly up-/downregulated when the absolute fold change (relative to the control) values were greater than 1 with a false discovery rate of less than 0.05. The raw data for analysis were deposited in SRA (Sequence Read Archive, http://www.ncbi.nlm.nih.gov/sra) with the accession number PRJNA113720.

### 2.9 Growth assay

To observe the growth rate of the wild-type (WT) and mutant strains, the strains were cultured in 5 mL of LB broth and incubated in an incubator shaker at 28°C and 180 rpm. The optical density (600 nm) was determined every 1 h over a period of 13 h.

### 2.10 Swarming and swimming assay

A single colony of the WT and mutant strain was inoculated in LB broth and overnight cultures were normalized to OD_600_ = 1. The bacterial suspensions (10 μL) were spotted in the center of LB agar (containing 0.7% agar) swarming plates to observe swarming motility. After 24 and 72 h of incubation, the migrated distances of bacterial colonies were measured. The means of three independent measures were calculated and accessed. For the swimming assay, overnight cultured bacterial cells (2 μL) were inoculated on LB agar plates (containing 0.3% agar).

### 2.11 Biofilm formation assay

Biofilm formation was assessed as described previously (Lee et al., 2009b). Single bacterial colonies were picked from LB agar plates, transferred to fresh LB broth, and cultured overnight in an incubator shaker at 28°C (180 rpm). The bacterial concentration was adjusted to OD_600_ = 1. The bacterial suspensions were inoculated in a fresh LB broth at a dilution rate of 1:100, and 200 μL of this culture was added to each well of a 96-well plate, which was then incubated at 28°C for 72 h. The supernatant containing unattached bacteria was gently removed, and the plate was washed with distilled water. The biofilm was stained with 200 μL of 1% crystal violet for 5 min, and thereafter, the plate was washed thrice with water. Then, 100 μL of 33% glacial acetic acid was added to dissolve crystal violet. After 20 min, OD_590_ was measured to determine the biofilm formation ability of the bacteria. There were three biological and three technical replicates for this assay.

### 2.12 Stress resistance assays

Stress resistance assays were performed as previously described (Tang et al., 2019) with minor modifications. In brief, the WT and mutant strains of *P. plecoglossicida* were incubated in LB broth at 28°C for 24 h in order for the bacterial cultures to reach a stationary phase (OD_600_ = 2.0). The bacterial cells were collected by centrifugation (6,000×*g*, 10 min), washed twice with 0.2 M phosphate buffer (pH 7.0), and then diluted to a concentration of 10^7^ cfu/mL with the same buffer. The bacterial cultures were treated with 20 mM H_2_O_2_ or 30% (m/v) NaCl, or at a 45°C water bath, respectively. After 0, 10, 20, 30, and 40 min of treatment, surviving bacteria were detected by plating the treated cultures on fresh LB agar plates. The viable cell count was measured after 48 h of incubation at 28°C.

### 2.13 Statistical analysis

*t*-test was used to determine if the changes in growth, motility, and biofilm formation data were significant. One-way analysis of variance was used for multi-group comparison of gene transcription data. Statistical analysis was performed using SPSS v17.0 statistical software (SPSS Inc., Chicago, IL, USA). *p*-values < 0.05 were considered statistically significant.

## 3 Results

### 3.1 *P. plecoglossicida* NB2011 LuxR family protein and proteins from other bacterial strains

**Figure 1:**
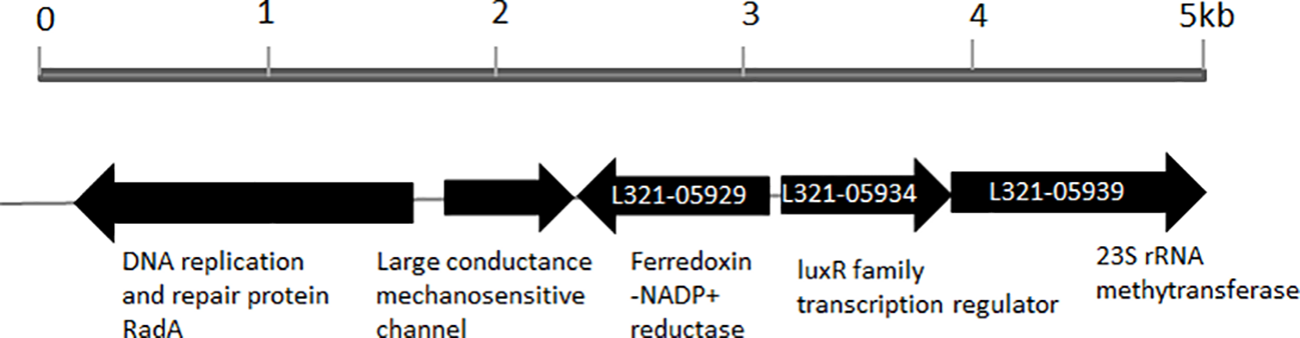
Physical map of *luxR solo* gene in the genome of *P. plecoglossicida* NB2011. The arrows indicate the coding region of *luxR solo (pplR)* of *P. plecoglossicida* NB2011 and a number of the adjacent genes were predicted by the JGI blast genome database (https://img.jgi.doe.gov/). The sequencing numbers of the target sequence and the adjacent genes were L321_05934, L321_05929, and L321_05939.

When the amino acid sequence of LuxR of *Vibrio harveyi* (accession no. AAN86705.2) was used as a subject to search the genome sequence of *P. plecoglossicida* NB2011 in JGI (https://img.jgi.doe.gov/), a sequence annotated as an autoinducer binding domain-containing protein and a LuxR family transcriptional regulator was found and designated as PplR. As shown in **Figure 1**, a 708-bp-long gene is located adjacent to the sequence encoding ferrodoxin-NADP^+^ reductase and 23S rRNA methytransferase without a predicted *luxI* gene upstream or downstream of the sequence.

**Figure 2:**
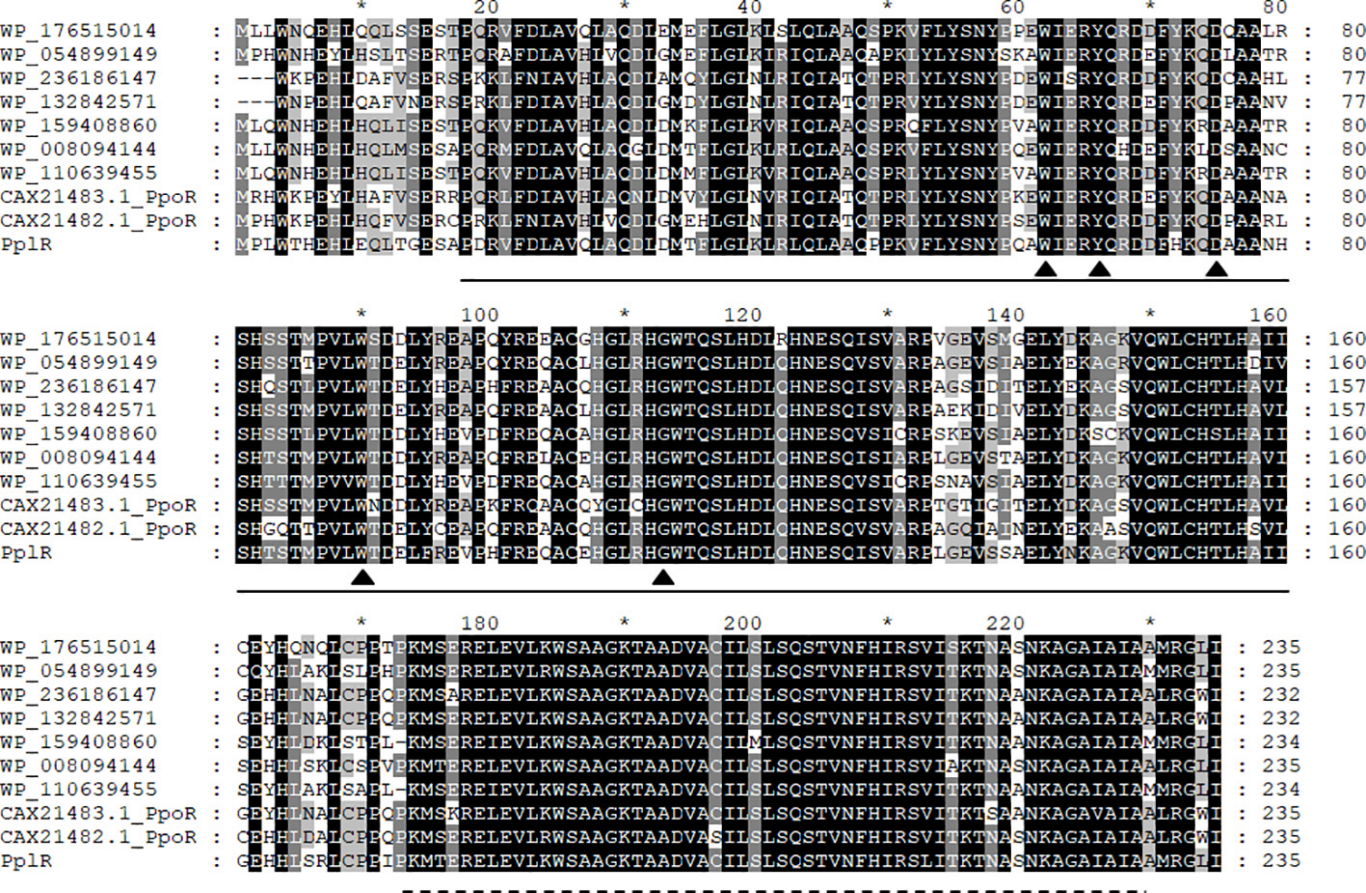
Multi-sequence alignment of PplR of *P. plecoglossicida* NB2011. Multiple sequence alignment was performed using CluxtalX2. The protein sequences used for alignment are as follows: *Pseudomonas putida* (CAX21482.1), *P. putida* (CAX21483.1), *P. plecoglossicida* NB2011 (PplR), *Pseudomonas* sp. BML-PP048 (WP_176515014), *Pseudomonas* sp. NBRC111131 (WP_054899149), *P. juntendi* (WP-236186147), *P. putida* (WP_132842571), *P. putida* (WP_159408860), *Pseudomonas* sp. GM84 (WP-008094144), and *Pseudomonas* sp. CC120222-01a (WP_110639455). Conserved amino acids are shaded in black; gray shading indicates that 100% of the residues are similar at that position. Also indicated are the regions of PplR of *P. plecoglossicida* NB2011 that constitutes the alto inducer binding domain (bold line from 18 to 160 amino acids) and the DNA binding domain (dashed line from 174 to 229 amino acids). The triangle indicates conserved residues essential for AHL binding and recognition. The symbol "*" indicates a number 10 less than the following number.

**Figure 2** presents the aligned translated amino acid sequence. As shown in **Figure 2**, homologous LuxR family transcription regulators are widely distributed in *Pseudomonas*, especially in *P. putida* and its relative species. The blasting of the sequence revealed two conserved domains—an N-terminal autoinducer binding domain (residues 18–160) that binds signal molecules specifically and a C-terminal DNA-binding domain (residues 174–229) that contains a helix-turn-helix (HTH) motif—that shared the conserved motif of W_62_Y_66_D_75_ W_90_G_113_, which is essential for AHL recognition and binding (Bez et al., 2021).

### 3.2 AHL detection and binding

AHL secretion by *P. plecoglossicida* NB2011 was tested by crossing feeding test and HPLC-MS/MS. No colored bacterial lawns were developed in the tests (data not shown), implying inactive pigment production in both reporter strains. The LC-MS/MS assay failed to detect a certain range of AHLs with expected molecular weight in the supernatant of either WT or mutant strain (data not shown), suggesting that the bacteria did not secrete AHL signal molecules.

**Figure 3:**
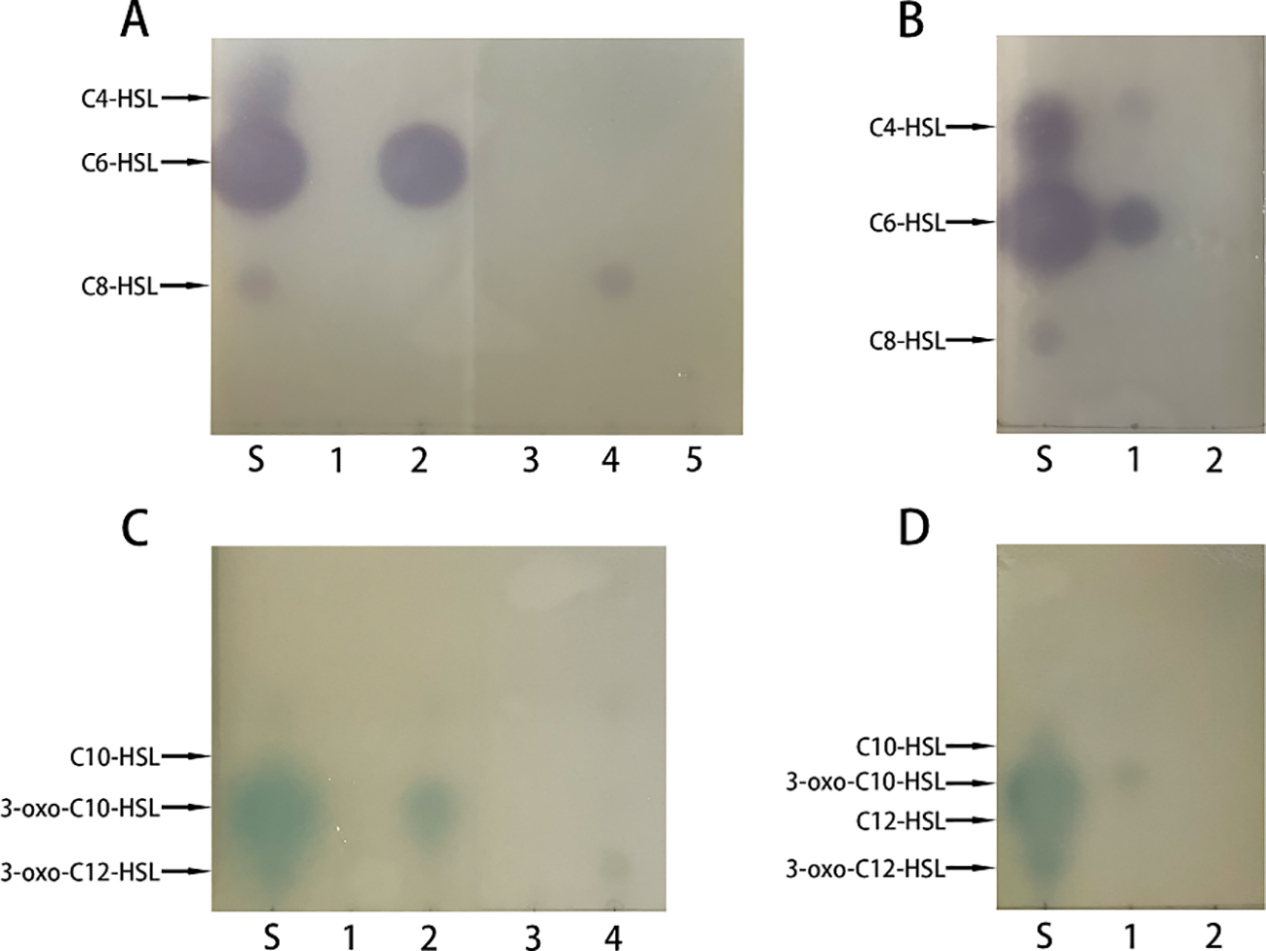
Recombinant PplR binds AHL signal molecules. *E. coli* BL21 containing either pSmart-*luxR* or pSmart-I were grown in LB in the presence of various AHLs (1 μM) added separately and protein expression was induced with IPTG (0.6 mM). After 4 h of growth, AHLs were extracted from the cell pellets and visualized by TLC overlaid with *C. violaceum* CV026 **(A, B)** or *A. tumefaciens* KYC55 **(C, D)**. The standards were synthetic AHLs. The lanes are marked as follows: S, AHL standards, A1–5, AHL extraction from *E. coli*/pSmart-*pplR* cell pellets grown with C4-HSL, C6-HSL, 3-oxo-C6-HSL, C8-HSL, and 3-oxo-C8-HSL; C1–4, AHL extraction from *E coli*/pSmart-*pplR* cell pellets grown with C10-HSL, 3-oxo-C10-HSL, C12-HSL, and 3-oxo-C12-HSL; B1 and D1, AHLs extraction from *P. aeruginosa* ATCC27853; B2 and D2, AHL extraction from *E. coli*/pSmartI cell pellets grown with C6-HSL and 3-oxo-C10-HSL.

To check whether the recombinant PplR binds AHLs, a successful expression of the target protein by *E. coli* BL21 (pSmart-*pplR*) was induced. Thereafter, the binding of exogenous AHLs by *E. coli*-expressed PplR was tested by TLC with *C. violaceum* CV026 or *A. tumefaciens* KYC55 (**Figure 3**). Purple or blue spots were developed with AHL extraction from the cell pellets of *E. coli*/pSmart-*pplR* supplemented with C6-HSL, C8-HSL (**Figure 3A**), 3-oxo-C10-HSL, and 3-oxo-C12-HSL (**Figure 3C**). While a much darker spot was observed with C6-HSL, pigmentation appeared a little lighter with the other three AHLs, indicating a broad binding spectrum of the protein and the strongest binding affinity with C6-HSL. As a positive control, AHL synthesis in *P. aeruginosa* ATCC27853 was determined (**Figures 3B, D**); three signals—C4-HSL, C6-HSL, and 3-oxo-C10-HSL—were detected in the supernatant. No pigmentation was developed with the AHL extracts obtained from the cell pellets of *E. coli*/pSmart supplemented with C6-HSL (**Figure 3B**), 3-oxo-C10-HSL (**Figure 3D**), and seven other AHL standards (data not shown).

### 3.3 DEGs in *pplR* mutant and the WT strain

RNA-seq-based transcription analysis revealed 84 differentially expressed genes (DEGs)—mainly downregulated (79) and a few upregulated (5)—in the mutant strain as listed in **Table 2**. Three genes involved in flagella-dependent motility were upregulated. Among downregulated DEGs, the most enriched GO terms were integral component of membrane (six genes), DNA binding (four genes), metal ion binding (four genes), and regulation of DNA-templated transcription (three genes). The other terms showed no apparent enrichment as only one or two such DEGs were identified and classified. The sequence of the autoinducer binding domain-containing protein, L321_RS05800 (corresponding to the gene of *pplR*, L321-05934), was successfully deleted from the bacterial genome; the expression of this gene was not detectable in the mutant strain.

**Table 2:** DEGs in *pplR* mutant compared to the wild-type strain.

Gene ID	Gene name	Fold change log2FC	*p*-value	*p*_adjust_	Annotation
Flagellar-dependent motility					
L321_RS14665	fliE	1.019963	0.000127	0.010839	Flagellar hook-basal body complex protein FliE
L321_RS14805	flgC	0.977132	0.000283	0.01973	Flagellar basal body rod protein FlgC
L321_RS14780	flgG	0.803783	0.000827	0.041722	Flagellar basal-body rod protein FlgG
Integral component of membrane					
L321_RS05230		−1.16809	0.000118	0.010691	DUF-2946 domain-containing protein
L321_RS21465		−1.27247	5.52E-07	0.000132	DUF4381 domain-containing protein
L321_RS04700		−1.34049	6.78E-08	2.80E-5	Hypothetical protein
L321_RS08160	PnuC	−1.76327	8.41E-07	0.000182	Nicotinamide riboside transporter pnuC
L321_RS16955		−1.1105	6.78E-07	0.000154	Hypothetical protein
L321_RS19145		−1.09927	1.36E-06	0.000258	MFS transporter
DNA binding					
L321_RS16900	Zur	−1.16736	1.42E-07	4.59E-5	Zinc uptake transcriptional repressor Zur
L321_RS20560		−1.27878	2.63E-09	1.49E-6	Sigma-70 family RNA polymerase sigma factor
L321_RS03250		−1.17279	4.58E-07	0.000115	Response regulator transcription factor
L321_RS05800		−9.34398	2.00E-33	9.08E-30	Autoinducer binding domain-containing protein
Metal ion binding					
L321_RS06675		−1.61367	1.74E-07	5.26E-5	Methyltransferase domain-containing protein
L321_RS19785		−1.01369	0.000784	0.040441	ABC-transporter substrate-binding protein
L321_RS16900	Zur	−1.16736	1.42E-07	4.59E-5	Zinc uptake transcriptional repressor Zur
L321_RS08870		−1.42264	2.73E-08	1.49E-6	Zn-dependent hydrolase
Regulation of DNA-templated transcription					
L321_RS15330	norR	−1.12115	2.23E-05	0.00274	Nitric oxide reductase transcriptional regulator NorR
L321_RS03250		−1.17279	4.58E-07	0.000115	Response regulator transcription factor
L321_RS05800		−9.34398	2.00E-33	9.08E-30	Autoinducer binding domain-containing protein
Secretion system					
L321-RS19105	tagF	−1.1540	2.5491	0.0001	Type VI secretion system-associated protein TagF

### 3.4 WT and mutant strain growth patterns

**Figure 4:**
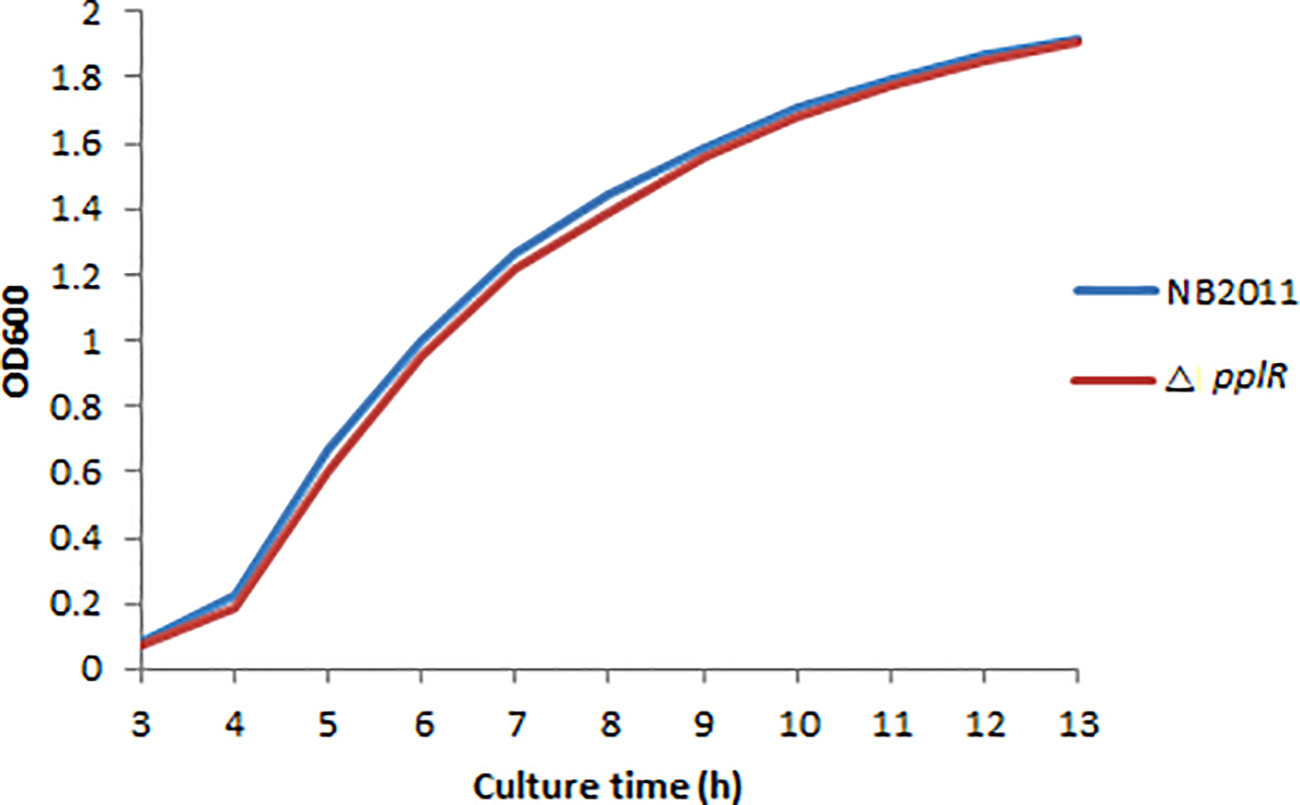
Growth curves of WT and mutant strains. The bacterial concentration was determined by measuring the optical density at 600 nm within 13 h, and the growth curve diagram was drawn.

As shown in **Figure 4**, the deletion mutant showed a growth curve similar to that of the WT strain. No significant difference (*p* > 0.05) in growth was found during lag and exponential phases of the two bacterial strains, indicating that the target gene does not directly affect bacterial growth.

### 3.5 Swimming and swarming motility of the mutant

**Figure 5:**
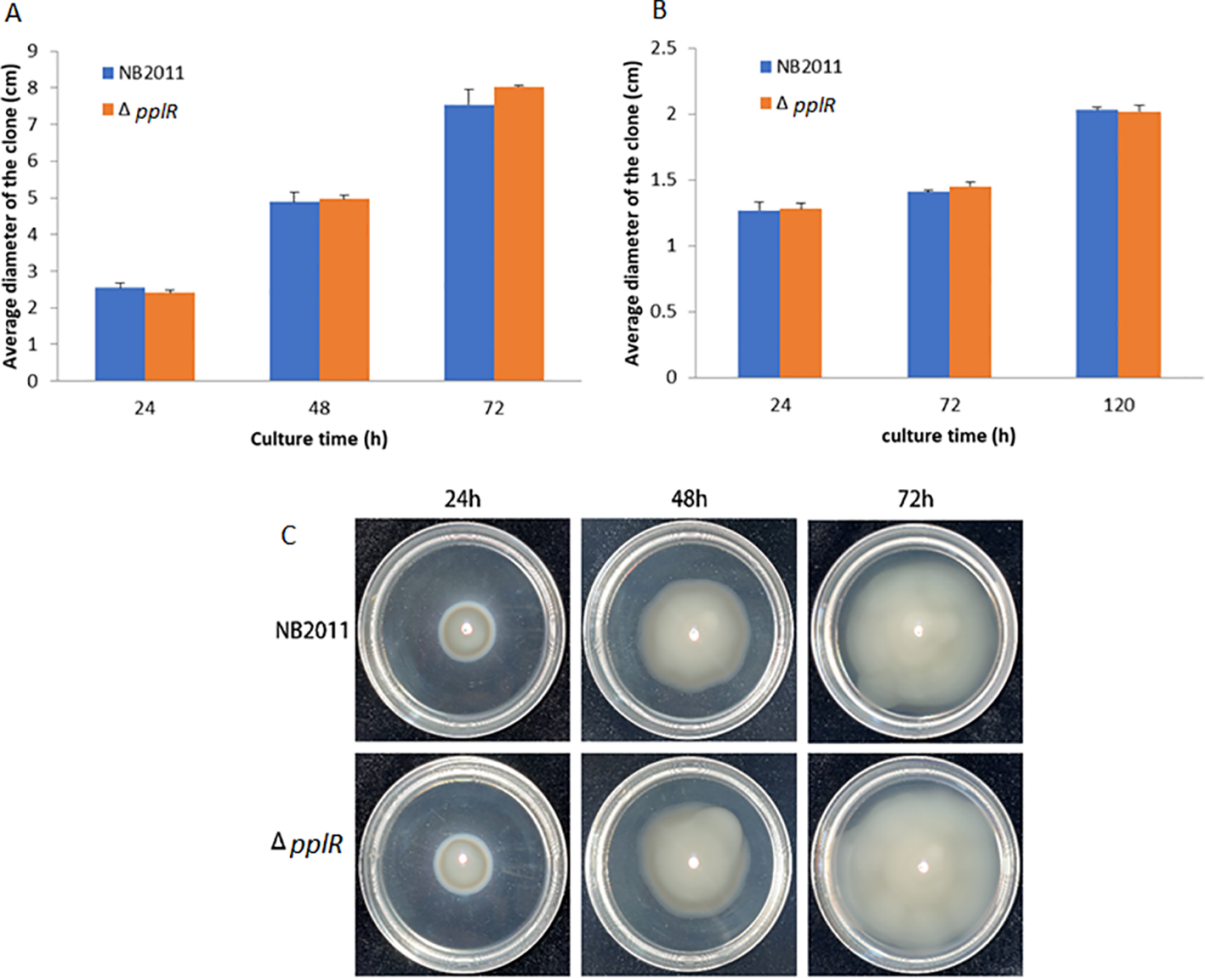
Swimming and swarming motility of mutant and wild-type strains. **(A)** Swimming motility of mutant and WT strains after 72 h. **(B)** Swarming motility of the mutant and WT strains after 120 h. **(C)** Pictures of the representative clones from the swimming test after 72 h.

As shown in **Figures 5A, C**, during the first 48 h, the swimming motility of the two strains appeared the same and the average diameter of the clones was very close. At 72 h, the mutant strain swam a little faster than the WT strain, although the difference was not statistically significant (*p* > 0.05). The swarming motility of the WT and mutant strain appeared similar during the entire experimental duration (0–120 h) (**Figure 5B**).

### 3.6 Biofilm formation of WT and mutant strains with or without AHL extracts

**Figure 6:**
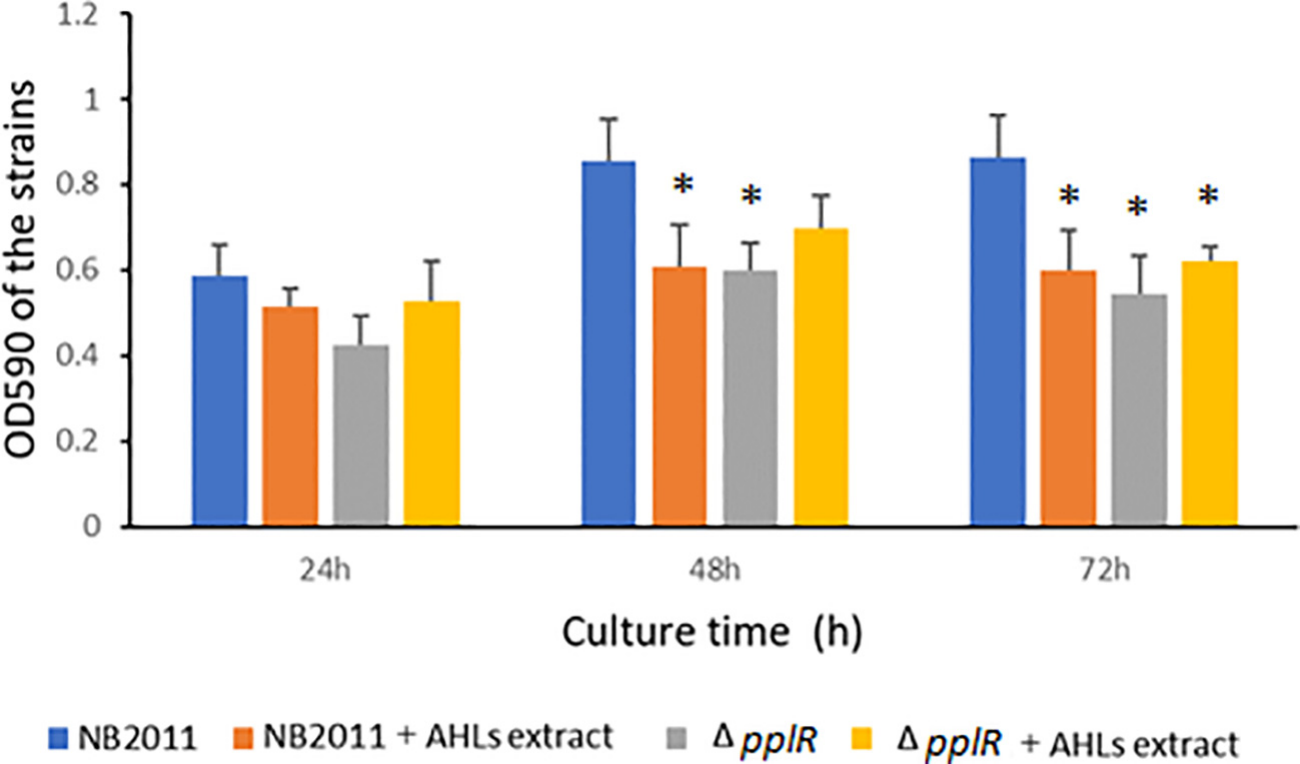
Biofilm formation of the wild-type and mutant strain with or without AHL extract from *P. aeruginosa*. Data are presented as mean ± SD of three independent experiments. “*” indicated significant differences (*p* < 0.05) between WT and other strains.

The biofilm formation capability was tested during 24–72 h of bacterial growth. As shown in **Figure 6**, the biofilm formation ability of the WT and mutant strains increased from 0 to 48 h independent of the presence or absence of AHL extract. During the following 24 h, the biofilm formation ability of the WT strain was stable, whereas the ability of the mutant strain slightly declined. In the absence of AHL extracts from *P. aeruginosa* ATCC27853, the biofilm formation ability of the mutant strain was lower than that of the WT strain during the entire experimental period. This difference was significant at 48 and 72 h (*p* < 0.05), indicating the attenuation of biofilm formation due to the deletion of *pplR*. When the AHL extract was provided, biofilm formation reduced in the WT but not in the mutant, and the difference was significant at 48 and 72 h (*p* < 0.05), indicating that biofilm formation was repressed by AHLs secreted by *P. aeruginosa*, and this action was *pplR* dependent. In comparison to the mutant strain without AHL extract from *P. aeruginosa*, a slightly higher OD_590_ was observed in both WT and mutant strains supplemented with AHL extract, although the difference was not significant (*p* > 0.05), implying that other signals that stimulate biofilm formation do exist in the extract independent of the presence of *pplR* and AHL signal molecules.

### 3.7 *PplR* deletion reduces stress resistance

**Figure 7:**
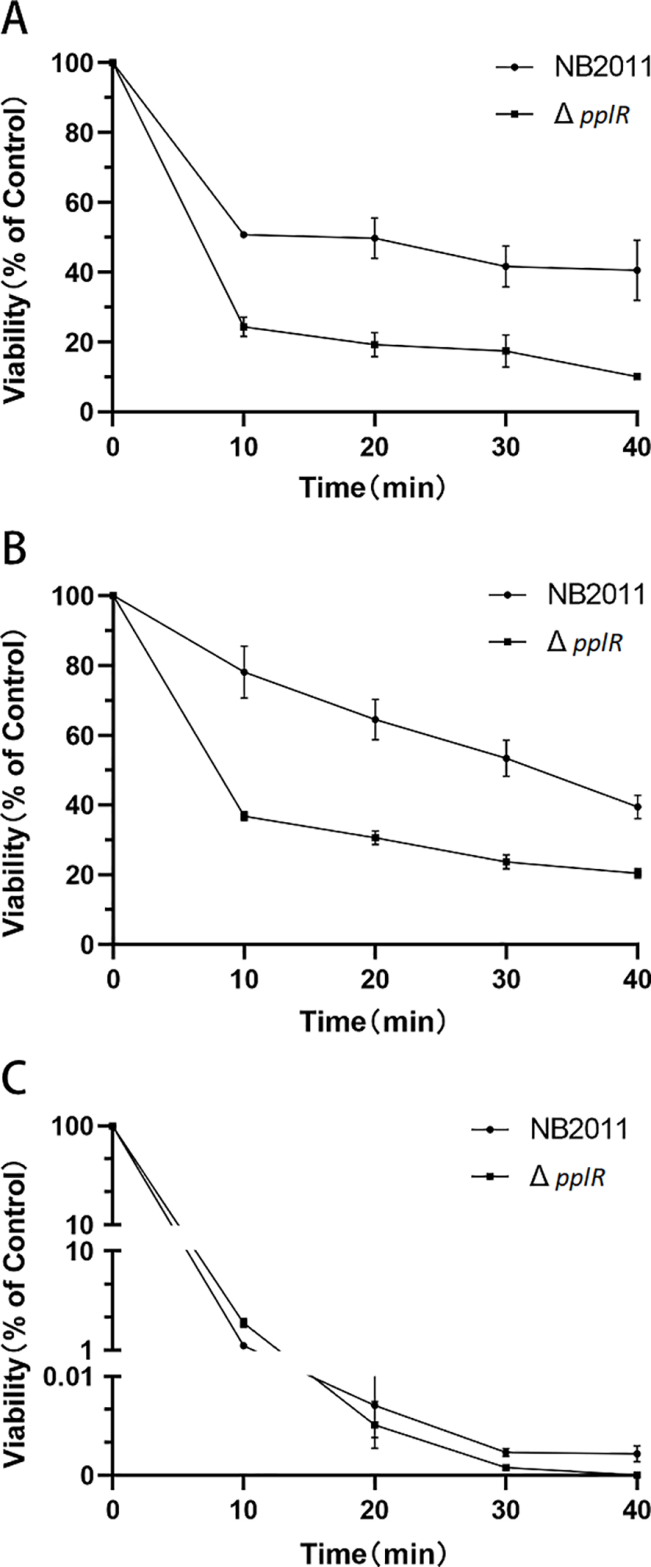
Survival rates of WT and mutant strains after H_2_O_2_, NaCl, and thermal stress. **(A–C)** Survival rates of the strains exposed to 20 mM H_2_O_2_, 30% NaCl, and thermal stress (45°C), respectively. The initial population of *P. plecoglossicida* NB2011 was 10^7^ cfu/mL. Survival percentage was calculated by dividing the surviving population by the initial population, which corresponds to 100%. Data are presented as mean ± SD of three independent experiments.

The survival rates of WT and Δ*pplR* strain in the stationary phase were determined after exposure to various stress conditions, such as H_2_O_2_, NaCl, and thermal. As shown in **Figure 7**, the mutant exhibited lower survival rates than the WT cells. When treated with 20 mM H_2_O_2_, the survival rates of the WT and mutant strains declined drastically in the first 10 min; 24.3% and 50.7% of the mutant and WT strains survived, respectively. During the following 30 min, the WT cells appeared stable with a survival rate of 40.6% at the end of the experiment; however, the number of mutant cells decreased gradually with a survival rate that declined to 10.1% at the end of the experiment. The survival of the mutant treated with 30% NaCl declined sharply in the first 10 min and then declined gradually to 20.4% at the end of the experiment. However, the number of viable WT cells declined continuously during the entire experimental period with a survival rate of 39.5% after 40 min. After thermal treatment of the bacteria, the survival rates of WT and mutant strains declined in the first 30 min. A few WT cells survived at the end of the experiment; while the mutant cells were not detected toward the end of the treatment (**Figure 7C**).

## 4 Discussion

### 4.1 LuxR solo homolog classification in *P. plecoglossicida* NB2011

*LuxR solo* without a cognate *luxI* is frequently found in Gram-negative bacteria. One to three orphan *luxR* are present in these strains. Researchers have classified Proteobacterial LuxR solos into five groups, including LuxR solos responding to endogenous AHLs (A group), such as QscR, exogenous AHLs (B group), such as SdiA and PpoR, non-AHL endogenous signals (C group), non-AHL exogenous signals (D group), and LuxR solos acting in a ligand-independent manner (E group) (Bez et al., 2023). In environmental fluorescent *Pseudomonas* species, nine groups of LuxR solo were identified, and the flank genes in the first group were 23S rRNA and ferrodoxin-NADP+ reductase (Bez et al., 2021). PpoR of *P. putida—*a conserved LuxR solo belonging to the first group*—*binds to exogenous 3-oxo-C6-HSL (Subramoni and Venturi, 2009) or C6-HSL (Lee et al., 2010). PplR, LuxR solo of *P. plecoglossicida* NB2011 that shared high similarity with PpoR, which lies adjacent to 23S rRNA and ferrodoxin-NADP+ reductase, encoded conserved motifs for signal binding and recognition, and the protein may bind AHL signal molecules and function similarly.

### 4.2 *P. plecoglossicida* recombinant PplR binds to AHLs with N-acyl side chains from C6 to C12

In our studies, endogenous AHL production by *P. plecoglossicida* NB2011 was not observed; however, the recombinant PplR was found to bind C6-HSL, C8-HSL, 3-oxo-C10-HSL, and 3-oxo-C12-HSL, which confirms that the protein belongs to the LuxR solo group responding to exogenous AHL signal molecules. In comparing PpoR (in *P. putida*) binding to 3-oxo-C6-HSL and C6-HSL (Subramoni and Venturi, 2009; Lee et al., 2010), the protein showed strong binding affinity toward AHLs with a longer N-acyl chain, indicating a relaxed ligand-binding specificity to a variety of AHLs, similar to QscR (Papenfort and Bassler, 2016) and SdiA (Smith et al., 2011). Further studies are required to find more possible AHL signals for this protein. There are two conserved domains in the LuxR family proteins, including the N-terminal autoinducer binding domain and the C-terminal HTH domain for DNA binding. The specificity of autoinducer binding is determined by the N-terminal (Bez et al., 2021). The sequence difference between the N-terminals of the two proteins may account for diverse recognition of AHLs though they shared a high similarity (72.6%).

### 4.3 *P. plecoglossicida* PplR may bind to AHL extract from *P. aeruginosa* and may be involved in repressing biofilm formation

Furthermore, the observation that the AHL extract from *P. aeruginosa* repressed biofilm formation in the WT strain provided some cues that the protein should have bound to exogenous AHLs. *P. aeruginosa* isolates encode two different AHL QS systems*—*the Las and Rhl*—*and produce a variety of AHLs from C4 to C12-HSL (Vadakkan et al., 2024; Gowda et al., 2013). As evidenced by AHL binding assay, *P. aeruginosa* ATCC27853 produced three signals of C4-HSL, C6-HSL, and 3-oxo-C10-HSL. Supplementation with AHL extracts of this strain significantly reduced biofilm formation in the WT strain but not in the mutant strain, suggesting that the WT strain might have bound to exogenous AHLs, which were *pplR* dependent. These findings were in corroboration with the AHL binding results. Similar findings have been observed in PpoR of *P. putida*; the heterologous AHL signals bind to PpoR and repress biofilm formation (Lee et al., 2010). Nevertheless, a slight stimulation in biofilm formation was observed in the mutant strain, implying that other signals independent of LuxR exist in the *P. aeruginosa* extract. Indole is expected to alter gene expression extensively in a manner contrary to AHLs (Lee et al., 2009a). Similar phenomena were observed in PpoR of *P. putida* (Lee et al., 2009b). Based on these results, we assume two possibilities of LuxR functioning in our study—PplR of *P. plecoglossicida* NB2011 itself (or binding to a yet unidentified endogenous signal) acts as a positive transcription regulator of biofilm formation or LuxR solo binds to exogenous AHLs, and the LuxR–AHL complex negatively regulates transcription of biofilm formation.

It is worth mentioning that some molecules other than AHLs may be present to inhibit biofilm formation in this study, since no negative control of AHL-deficient *P. aeruginosa* extract has been used. To verify PplR responding to AHLs, an AHL-deficient *P. aeruginosa* should be applied, and in a further study, a luciferase assay for the promoter, or a gel shift assay with purified PplR in the presence or absence of AHL, should be carried out.

### 4.4 *P. plecoglossicida* PplR plays a role in regulating biofilm formation but not surface motility

Bacteria QS systems involved in biofilm formation and other group phenotypes responding to cell density have been well documented (Papenfort and Bassler, 2016). In our study, without exogenous AHLs, the *pplR* deletion mutant of *P. plecoglossicida* NB2011 produced weak biofilm, indicating that the protein plays a role in positively regulating biofilm formation in the bacteria, which is similar to PpoR of *P. putida* KT2440 (Lee et al., 2010) and a LuxR homolog in *P. fluorescens* PF07. The researchers suggested that *luxR* deficiency could inhibit the expression of exopolysaccharide matrix and induce flagella-driven motility by affecting the second signal messenger c-di-GMP, which represses the conversion of biofilm from planktonic cells (Tang et al., 2019). In contrast, when supplemented with AHL extracts from *P. aeruginosa*, repression of biofilm formation was observed, like sdiA of *Cronobacter sakazakii* and *K. pneumoniae*, which bound exogenous AHLs and suppressed biofilm formation (Pacheco et al., 2021). Cao and colleagues suggested that enhanced motility, decreased surface hydrophobicity, and cellular matrix may suppress biofilm formation (Cao et al., 2022). In *P. plecoglossicida* NB2011, stimulation or suppression of biofilm formation may occur under different situations. LuxR solo-promoted biofilm formation has been reported to play important roles in pathogenesis (Bez et al., 2023), which may facilitate bacterial colonization in the intestine of infected fish (Li et al., 2020). However, during inhabitation with other commensal bacteria in water, high exogenous AHL levels indicate intense competition for nutrition, dissolved oxygen, and living space. The LuxR–AHL complex suppresses biofilm formation but promotes a planktonic lifestyle to search more suitable environment for bacteria.

Regarding surface motility, in *P. putida* KT2440, PpoR enhances the swarming motility of the bacteria (Fernandez-Pinar et al., 2011). In *P. plecoglossicida* NB2011, similar swimming and swarming motility was observed in the *pplR* mutant and WT strain, indicating that the surface motility is not regulated by this protein. In general, bacteria have developed motility to exploit available resources and environments (Wadhwa and Berg, 2022). The discrepancy may lie in the lifestyles of the two bacterial species—*P. plecoglossicida* is a facultative pathogen of fishes (Mao et al., 2013a; Zhang et al., 2014), whereas *P. putida* KT2440 is a rhizosphere strain living with plants (Wu et al., 2011). A QS-regulated surface motility would greatly favor the bacteria in commensal environment.

### 4.5 *P. plecoglossicida* PplR plays a role in enhancing stress resistance in bacteria

With respect to stress resistance, the *pplR* mutant of *P. plecoglossicida* revealed diminished survival ability to H_2_O_2_, NaCl, and thermal stress. Similar results have been observed in the LuxI/LuxR mutants of *P. fluorescens* (Tang et al., 2019). In the PpoR++ strain of *P. putida* WCS 358 (containing pBBRPpoR), genes involved in oxidative resistance and inorganic ion utilization were significantly upregulated (Subramoni and Venturi, 2009). This suggests that the LuxR homologs are positive regulators of environmental stress in these species. In *P. aeruginosa*, the QS system regulates superoxide dismutase and catalase during oxidative stress (Hassett et al., 2010), and in *V. harveyi*, QS is linked to osmotic stress response (Van Kessel et al., 2015). Our observations imply that the LuxR solo of *P. plecoglossicida* may have a role in imparting resistance to oxidative and osmotic stresses. Considering the attenuated intracellular reproduction of the mutant in mouse macrophage J774A.1 (data not shown), enhanced resistance of *P. plecoglossicida* NB2011 to oxidative stress may play an important role in bacterial pathogenicity, thereby protecting the bacteria from not being killed by fish macrophages and successfully establishing infection in the croaker (Mao et al., 2013b). However, the exact mechanism behind the response should be explored in future studies.

### 4.6 PplR regulates major biological processes in *P. plecoglossicida*

RNA transcript analysis revealed significant DEGs, including upregulated DEGs involved in flagellar synthesis and downregulated DEGs enriched in DNA binding, regulation of DNA-templated transcription, integral component of membrane, and metal ion binding in the mutant strain. Downregulated T6SS-associated protein TagF may reduce T6SS activity, which is involved in biofilm formation in *P. plecoglossicida* NB2011 (Li et al., 2022), and this partially accounts for the decreased biofilm formation ability of the mutant. Although three flagella genes (FlgC, FlgG, and FliE) were found to be upregulated in transcription analysis, a significant increase in swimming motility was not observed in the present study. This finding was contrary to that of the *sdiA* mutant of *C. sakazakii*, which showed upregulated expression of FliA and FliC and increased swimming motility (Cheng et al., 2022). This suggests that there may be some other factors that negatively regulate flagellar motility in our studies. Like other LuxR solo family proteins (Bez et al., 2023), downregulation of DNA-binding and DNA-templated transcription in the present study suggested PplR as an important regulator of transcription. With regard to metal ion utilization, it was not tested in the present study.

It should be mentioned that the design of the RNA-seq experiment could be improved in further research; in the present study, the time point of sampling was 24 h post-culture; setting more time points may help to identify a clear connection between the phenotypes and DEGs, as evidenced by the difference in biofilm formation between the mutant and WT, with the culture time increased from 24 to 48 h.

## 5 Conclusions

In general, the data in the present study support a role for PplR, LuxR solo of *P. plecoglossicida* NB2011—a homolog of PpoR of *P. putida—*in regulating biofilm formation and stress resistance. The *E. coli*-expressed recombinant PplR binds to exogenous AHLs, such as C6-HSL, C8-HSL, 3-oxo-C10-HSL, and 3-oxo-C12-HSL. Like other LuxR family members being widely involved in QS-related phenotypes, PplR seems to be a global QS regulator involved in interspecies communication. As *P. plecoglossicida* NB2011 did not secrete AHLs as autoinducers of the QS system, the kind of signals that the strain produces is yet to be explored. Additionally, it needs to be understood what signals other than AHLs the LuxR solo responds to. Is the signal an as-yet-unidentified molecule that the researchers are not familiar with?—It is an open-ended question.

## Data Availability

The datasets presented in this study can be found in online repositories. The names of the repository/repositories and accession number(s) can be found in the article/supplementary material.
